# Focus and coverage of *Bolsa Família* Program in the Pelotas 2004 birth cohort

**DOI:** 10.1590/S1518-8787.2017051006792

**Published:** 2017-03-31

**Authors:** Kelen H Schmidt, Jeremy Labrecque, Iná S Santos, Alicia Matijasevich, Fernando C Barros, Aluisio J D Barros

**Affiliations:** I Programa de Pós-Graduação em Nutrição em Saúde Pública. Faculdade de Saúde Pública. Universidade de São Paulo. São Paulo, SP, Brasil; II Department of Epidemiology. Biostatistics and Occupational Health. McGill University. Montreal, Canadá; III Programa de Pós-Graduação em Epidemiologia. Universidade Federal de Pelotas. Pelotas, RS, Brasil; IV Departamento de Medicina Preventiva. Faculdade de Medicina. Universidade de São Paulo. São Paulo, SP, Brasil

**Keywords:** Poverty, economy, Government programs, provision & distribution, Wages and benefits, Income, Development indicators, Community development

## Abstract

**OBJECTIVE:**

To describe the focalization and coverage of *Bolsa Família* Program among the families of children who are part of the 2004 Pelotas birth cohort (2004 cohort).

**METHODS:**

The data used derives from the integration of information from the 2004 cohort and the *Cadastro* Único *para Programas Sociais do Governo Federal* (CadÚnico – Register for Social Programs of the Federal Government), in the 2004-2010 period. We estimated the program coverage (percentage of eligible people who receive the benefit) and its focus (proportion of eligible people among the beneficiaries). We used two criteria to define eligibility: the *per capita* household income reported in the cohort follow-ups and belonging to the 20% poorest families according to the National Economic Indicator (IEN), an asset index.

**RESULTS:**

Between 2004 and 2010, the proportion of families in the cohort that received the benefit increased from 11% to 34%. We observed an increase in all wealth quintiles. In 2010, by income and wealth quintiles (IEN), 62%-72% of the families were beneficiaries among the 20% poorest people, 2%-5% among the 20% richest people, and about 30% of families of the intermediate quintile. According to household income (minus the benefit) 29% of families were eligible in 2004 and 16% in 2010. By the same criteria, the coverage of the program increased from 43% in 2004 to 71% in 2010. In the same period, by the wealth criterion (IEN), coverage increased from 29% to 63%. The focalization of the program decreased from 78% in 2004 to 32% in 2010 according to income, and remained constant (37%) according to the IEN.

**CONCLUSIONS:**

Among the families of the 2004 cohort, there was a significant increase in the program coverage, from its inception until 2010, when it was near 70%. The focus of the program was below 40% in 2010, indicating that more than half of the beneficiaries did not belong to the target population.

## RESUMO

**OBJETIVO:**

Descrever a focalização e a cobertura do Programa Bolsa Família nas famílias de crianças que fazem parte da coorte de nascimentos de Pelotas, 2004 (coorte de 2004).

**MÉTODOS:**

Os dados utilizados derivam da integração de informações da coorte de 2004 e do Cadastro Único para Programas Sociais do Governo Federal, no período de 2004 a 2010. Estimamos a cobertura do programa (percentual de elegíveis que recebem bolsa) e seu foco (proporção de elegíveis entre os beneficiários). Utilizamos dois critérios para definir elegibilidade: a renda familiar *per capita* relatada nas avaliações da coorte e pertencer aos 20,0% mais pobres pela classificação do Indicador Econômico Nacional, um índice de bens.

**RESULTADOS:**

Entre 2004 e 2010, a proporção de famílias beneficiárias da coorte passou de 11% para 34%. Houve aumento em todos os quintis de riqueza. Em 2010, por quintis de renda e Indicador Econômico Nacional, 62%-72% das famílias eram beneficiárias entre os 20% mais pobres, 2%-5% entre os 20% mais ricos, e cerca de 30% das famílias do quintil intermediário. Pelo critério de renda familiar, excluindo-se o valor do benefício do programa, 29% das famílias eram elegíveis em 2004 e 16% em 2010. Pelo mesmo critério, a cobertura do programa passou de 43% em 2004 para 71% em 2010. No mesmo período, pelo critério de riqueza (Indicador Econômico Nacional), a cobertura passou de 29% para 63%. A focalização do programa caiu de 78% em 2004 para 32% em 2010 de acordo com a renda e permaneceu constante (37%) de acordo com o Indicador Econômico Nacional.

**CONCLUSÕES:**

Entre as famílias da coorte de 2004, observa-se aumento importante da cobertura do programa, de seu início até 2010, quando ficou perto de 70%. O foco do programa ficou abaixo de 40% em 2010, indicando que mais da metade dos beneficiários não pertencem à população alvo.

## INTRODUCTION

Brazil experienced a period of economic and social development between 2003 and 2013^a^. There was a great reduction in the poor and extremely poor population, an increase in the income of 40% poorest in the population, and a systematic reduction in income inequality^9^ ^,^ ^14^ ^,^ ^b^, with a decrease in the Gini index from 0.59, in 2001, to 0.54 in 2013^8^ ^,^ ^c^. Brazil reached the position of seventh greatest global economy in terms of growth of gross domestic product (GDP), with an increase in the average per capita household income from 8.1 to 12.1 dollars per day^9^. However, in the last four years, the situation has been changing, with reduction in the GDP growth rate (2.1% between 2011 and 2014, and 0.1% in 2014), high inflation (6.4% in 2014), decrease in exports, and increasing unemployment^d^. Measures were implemented to reduce the fiscal deficit, such as reducing benefits, cutback in expenses, and reduced support for public banks and the energy sector^9^. In relation to social indicators (health, child mortality, and nutrition), large regional differences are found. Inequality remains relatively high for a middle income country, with 8.9% of the population living in poverty and one-third of the population living in condition of economic vulnerability, without professional training and employability^9^.

Brazilian social development policies are not recent, and policies based on food distribution or subsidies for the purchase of necessity goods preceded cash transfer programs. The first of these was the *Programa Bolsa Escola* (PBE – School Assistance Program), created in 2001. At a municipal level, other attempts had already been implemented, but only in the second half of the 1990s this kind of social policy began to expand^e^.

In 2003, the *Bolsa Família* Program (BFP) was launched, unifying the existing programs (cooking gas, schooling and feeding benefits) and simplifying this structure. Its main goal was to eradicate extreme poverty and hunger^22^ ^,^ ^f^. Currently, to be eligible for the program, the family must have a monthly *per capita* income below R$154 (BRL), being classified as in extreme poverty if the *per capita* income is less than R$77 (BRL)^6^.

BFP offers unconditional cash transfer for extremely poor families and conditional cash transfer for poor families or extremely poor families that have children, young people under 18 years of age, pregnant women, or nursing mothers in their composition. The main BFP conditionalities are the adoption of preventive health habits, such as regular medical consultations, vaccinations, and anthropometric monitoring, and maintenance of a minimum level of school attendance of 85% of the school year, for children in elementary school, and 75% for young people in high school^6^.

To receive the assistance, which is normally granted on behalf of the mother of the family, it is necessary to be registered on *Cadastro Único para Programas Sociais do Governo Federal* (CadÚnico – Register for Social Programs of the Federal Government), which is managed by the Brazilian Ministry of Social Development (MDS). The application is made in service points of the municipality of residence, but the participation in the program is decided at a federal level, according to specific criteria and system availability. Having the application approved, holders of the benefit receive a debit card with which they can withdraw the benefit on a cash machine. The value of the benefit is the sum of a fixed amount and another which is variable according to the family composition (children, teenagers, pregnant women, or nursing mother)^22^.

BFP is a focused program, since eligibility is defined according to the *per capita* household income. Maintaining the focus of the program, that is, ensuring that most part of the intended resource is received by eligible families, is an important part of the management and ensures that the benefit does not “leak” to groups that, *a priori*, are not priority for the program. The use of focalization mechanisms in social programs is usually justified as a matter of “efficiency in the allocation of resources”, that is, it concentrates investment of a limited budget on people that are most in need^5^. Thus, according to Souza et al.^g^, focalization can be understood as an instrument to increase the coverage capacity of the program (percentage of eligible people who receive the benefit), considering the same amount of resources.

The methodology used by the MDS for defining the poverty line was based on an absolute criterion. Regionalized poverty lines were created based on calory consumption^19^, on the values used by World Bank and adopted by the United Nations, and the line proposed and used in studies of the Economic Commission for Latin America and the Caribbean (ECLAC). In the end, for the purpose of estimating and delimiting the priority public for BFP in 2010, the poverty line was established as a *per capita* income of R$140.00 (BRL), and the extreme poverty line at R$70.00 (BRL)^6^. Data from the Brazilian Institute of Geography and Statistics (IBGE, Census of 2010) used to estimate the size of the population in extreme poverty indicated that it was 16.3 million, about 8.5% of the total, concentrated in rural and Northern and Northeast regions^6^.

Some studies have evaluated the focus and coverage of BFP, at national or local level, being most of them performed with data from the *Pesquisa Nacional por Amostra de Domicílios* (PNAD – National Household Sample Survey), in different editions. From the 2004 PNAD data, a focus of 52% and 62% was reported for groups in extreme poverty and poverty, respectively, as well as a dramatically low coverage of approximately 14% for both groups^h^. Still using the 2004 and 2006 PNAD, coverage results between 70%-78% have been reported^14^ ^,^ ^c^. The program focus was between 51% and 58%^14^. A third study using PNAD 2004 data reported focus at 53% and 42% coverage^22^. With data from the 2010 Census, focus was 48% and coverage 59%^g^. The diversity of results shows the difficulty in establishing, in a robust way, which are the families that are part of the target group of the program. However, the results are consistent in the sense that both the focus and the coverage seem to be less than desirable.

One of the challenges in evaluating the focus of a national program such as BFP relates to the difficulty in collecting accurate income information, even by the program itself. On the other hand, surveys that collect income and household consumption more accurately do not collect information on the BFP, or have only very limited information. With detailed data on BFP participation for the families with children who are part of the 2004 Pelotas Birth Cohort, and with information on assets and education that allow estimation of the economic level of the families, we carried out a study aiming to describe the focalization and coverage of the BFP among the families of children who are part of this cohort.

## METHODS

This study used data from the 2004 Pelotas Birth Cohort (2004 cohort), including socioeconomic and family composition information. The 2004 cohort included 99% of live births to mothers residing in the urban area of the municipality of Pelotas, Rio Grande do Sul, during 2004, and the residents of Jardim América, current municipality of Capão do Leão (to maintain the same catchment area used for the 1982 Pelotas birth cohort). These children were evaluated at birth, with three, 12, and 24 months and with four and six years of age, when health-related information was recorded and anthropometric measurement were made. In the follow-up conducted between October 2010 and August 2011, when children were aged between six and seven years, we managed to enroll 90% of the original cohort (n = 3,721). The data used in this study combines information gathered at the various follow-up waves of the 2004 cohort. Details of the cohort methodology are described elsewhere^2^ ^,^ ^20^.

We also used data from CadÚnico and *Portal da Transparência* (Transparency Portal)^i^. CadÚnico data were obtained from the *Secretaria de Avaliação e Gestão da Informação* (MDS-SAGI – Department of Evaluation and Management of Information), comprising information on identification of beneficiaries, amounts monthly paid as well as some features of the house and the family covering the period 2004-2010. In addition, we collected information on participation in the BFP and monthly values received in the 2004-2010 period, taken from the site of *Portal da Transparência* on December 20, 2013.

Data from the three sources were integrated into a single dataset by a deterministic linkage process and, later, by a probabilistic one^6^. Keys to link the datasets were generated based on the child’s name, date of birth, name and age of the mother, and also the name of the father and grandmother of the child, since they were the beneficiaries of the program in some cases. The linkage process was performed with R software (23R Core Team, 2014). Initially, 4,231 children were evaluated in the cohort. The integration of databases allowed identifying 1,796 of these beneficiaries of BFP in the 2004-2010 period; of these, 1,494 (83.2%) had exactly the same information in the datasets.

Using this single dataset with complete information on each child, we assessed which children of the 2004 cohort were beneficiaries of BFP in each year, from 2004 to 2010, as well as in which months they received the benefit and the amounts received. We also used the information on BFP participation reported by the mothers at the six-year-old follow-up.

According to the proposal by Habitch et al.^8^, we defined program coverage as the percentage of eligible families which are beneficiaries of the program. Program focus was defined as the percentage of eligible families among all the beneficiaries. Symmetrically, we define leakage as the proportion of non-eligible people who are beneficiaries. It is important not to confuse the coverage of the program with the proportion of the population benefiting from the program, which is the percentage of families covered in relation to the total population, an indicator that does not consider whether the family is eligible or not.

To define eligible families, we used two indicators of economic classification in the analyses: the *per capita* household income (minus the value of the benefit received) and the National Economic Indicator (IEN). We used both criteria as a form of sensitivity analysis, in such a way to produce a plausible range of results, since the quality of information on income in surveys has been criticized for being heavily subject to errors of information and temporal variability^11^. Hence, we assume that these are real problems, but that there is no interest from the poorest families in hiding their income, since eligibility for the benefit is not in question. We also used an economic indicator based on household assets and on education of the household head, the IEN^1^, which is less subject to temporal variability because it is based on information that don’t change in short periods of time. The IEN is considered an indicator of permanent income, but it can also be subject to misclassification.

Therefore, for the *per capita* income criterion, we used the household income declared in the 2010 follow-up and the number of residents in the house to estimate the monthly average *per capita* income. In this analysis, we used as cut-off points for eligibility the values defined by the MDS – *per capita* income of R$100.00 in 2004 and R$140.00 in 2010^16^, excluding the value of the benefit received (estimated based on the monthly average of each year from CadÚnico data). In 2004, 15% of the households did not have complete information on income. We conducted a single imputation process for these values, from a linear regression model using the IEN variables plus whether the mother lives with a husband or partner, mother’s education, number of ultrasound examinations during pregnancy, and number of residents in the house. This model presented an R^2^ of 67%.

For the classification based on the IEN, the eligibility criterion was to belong to the 20% poorest families according to data from 2004 and 2010. This is a definition a little more flexible, since the indicator is not based on income, but produces a ranking of socioeconomic position based on the possession of household assets. The choice of 20% as cut-off point was based on the estimate, at the beginning of the program, that about 18% of the Brazilian population was classified as poor and, therefore, were potential beneficiaries of the program^21^. In addition, the BFP currently serves about 13,8 million families across the country, which corresponds to approximately 20%-25% of the Brazilian population^4^. Finally, a series of studies on poverty and inequality uses the cut-off point of 20% poorest to define relative poverty^1^ ^,^ ^12^ ^,^ ^13^ ^,^ ^18^ ^,^ ^19^ ^,^ ^j^.

We used standard techniques of descriptive analysis, presenting the results in terms of measures of central position and dispersion. Initially, we described the socioeconomic situation of the families presenting averages and standard deviations, overall and stratified by beneficiary status. Then, we identified the proportion of eligible families and the proportion of beneficiaries according to what was reported in the 2010 follow-up cohort and to the linkage dataset, by eligibility criteria (*per capita* income and IEN). Next, also using the two eligibility criteria, we estimated the coverage and focalization of the program in 2004 and 2010. We present the percentages and their respective 95% confidence intervals to allow an assessment of the accuracy of these estimates, as well as of the difference in proportions between subgroups. All analyses were carried out using the Stata software (StataCorp. 2013. Stata Statistical Software: Release 13. College Station, TX: StataCorp LP). All the follow-ups of the 2004 cohort were approved by the Research Ethics Committee of the Federal University of Pelotas Medical School. The use of data identified in CadÚnico has been approved and authorized by an MDS internal process. In all cases, we guarantee confidentiality and anonymity to the individuals involved.

## RESULTS

Table 1 shows sociodemographic characteristics of the 2004 cohort families, overall and by beneficiary status, for 2010. Beneficiary families of BFP presented a lower mean for income and asset score (IEN). These families are bigger and include less educated mothers than non-beneficiary families. There were no differences regarding maternal age at child’s birth.

**Table 1:** Characterization of the families of the 2004 Birth Cohort of Pelotas, by situation of beneficiary of the *Bolsa Família* Program, according to data from *Cadastro Único* and *Portal da Transparência* for 2010.

Characteristic	All (n = 3,639)		BFP Beneficiary			

			Yes (n = 1,245)		No (n = 2,394)	
		
	Average	Standard deviation	Average	Standard deviation	Average	Standard deviation
Household income (R$)	1,871.5	2,680.9	950.5	660.8	2,351.2	3,167.4
*Per capita* income (R$)	673.9	981.3	293.5	246.6	871.7	1148
*Per capita* income minus the benefit (R$)	662.9	985.5	261.5	244.5	871.7	1148
IEN (scores)	593.7	216.6	465.9	151.8	660.2	215.5
No. of residents in the house	3.3	1.4	3.8	1.6	3.1	1.3
Mother’s education (years of schooling)	8.7	3.7	6.7	3.1	9.7	3.7
Age of mother at birth (years)	26.1	6.8	26.0	6.7	26.1	6.8

BFP: *Bolsa Família* Program; IEN: National Economic Indicator, indicator of wealth based on household assets

The average value of the benefit received by the families was R$107.00 in 2010, equivalent to a monthly *per capita* value of R$32.00.

**Figure 1:**
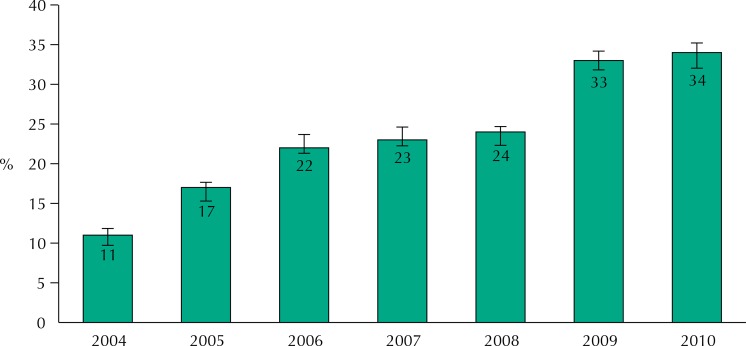
Proportion and confidence interval of beneficiaries of *Bolsa Família* Program among families of children of the 2004 Birth Cohort of Pelotas, according to data from *Cadastro Único* and *Portal da Transparência* for the 2004-2010 period.

The proportion of beneficiary families among participants of the 2004 cohort tripled between 2004 and 2010, increasing from 11% to 34% (Figure 1). There was an increase in the proportion of beneficiaries between 2004 and 2006, with stabilization until 2008, and further increase in 2009.

**Figure 2:**
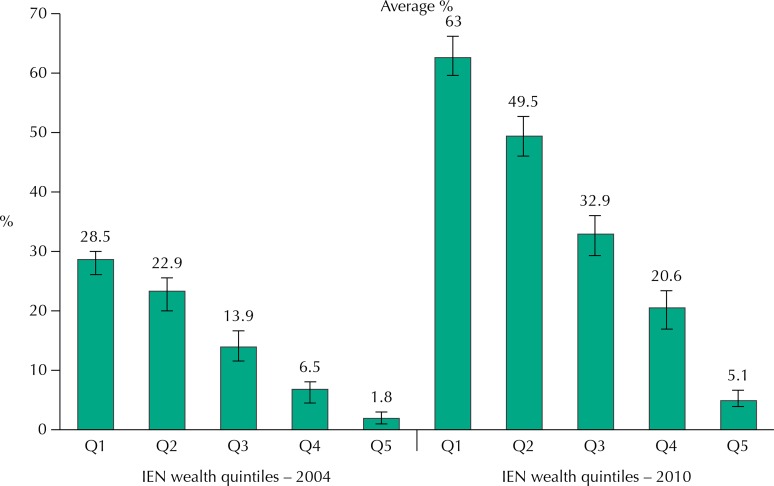
Proportion and confidence interval of beneficiaries of *Bolsa Família* Program among the families of children of the 2004 Birth Cohort of Pelotas, according to data from *Cadastro Único* and *Portal da Transparência*, by quintile of wealth (IEN = National Economic Indicator, from Q1 = 20% poorest to Q5 = 20% richest) for the 2004-2010 period.

Figure 2 shows the proportion of beneficiary families for 2004 and 2010, by IEN quintiles according to CadÚnico records. Instead of an abrupt fall in the percentage of families receiving the benefit with the increase in wealth, we verified a gradual decline, almost linear, from the first quintile (the 20% poorest) to the fifth quintile (20% richest). Between 2004 and 2010, the pattern remains the same, with a higher proportion of beneficiaries in all wealth quintiles.

In the 2010 follow-up of the 2004 cohort, when the children were aged six, we asked whether the family received the *Bolsa Família* benefit. The reported proportions of beneficiaries, overall and by wealth quintiles, are presented in Table 2, along with the proportion of beneficiaries identified from CadÚnico. The results are consistent, regardless of the data source, showing about one-third of the families receiving the benefit, with proportions, by income or IEN quintile, ranging from 62% to 72% among the poorest and from 2% to 5% among the richest. Again, we observe a gradual reduction in the proportion of beneficiaries from the poorest to the richest quintiles, with about 30% of families of the intermediate quintile receiving the benefit. The inspection of the confidence intervals, which do not overlap, shows that the differences between quintiles are all statistically significant.

**Table 2:** Percentage of beneficiaries of the *Bolsa Família* Program in the 2004 Birth Cohort of Pelotas in 2010 (based on information of *Cadastro* Único and on reports in the cohort questionnaire) by *per capita* household income quintiles (RFPC) and National Economic Indicator quintiles (IEN).

Quintile	*Per capita* household income					National Economic Indicator				
	
	Cut-off point (Brazilian currency)^a^	Beneficiaries				Cut-off point (score)^a^	Beneficiaries			
	
		*Cadastro Único* (n = 3,639)		Report (n = 3,631)			*Cadastro Único* (n = 3,651)		Report (n = 3,644)	
			
		%	95%CI	%	95%CI		%	95%CI	%	95%CI
Q1	0 -	71.9	68.6-75.2	69.9	66.5-73.2	64-	63.0	59.5-66.5	61.7	58.2-65.2
Q2	145 -	51.7	48.1-55.4	50.5	46.8-54.1	399-	49.5	45.9-53.2	48.7	45.0-52.3
Q3	307 -	28.4	25.1-31.7	23.8	20.7-26.9	519-	32.9	29.5-36.3	29.5	26.1-32.8
Q4	506 -	15.5	12.9-18.2	11.4	9.1-13.7	641-	20.6	17.6-23.5	14.5	12.0-17.1
Q5	771 -	3.4	2.1-4.8	2.1	1.0-3.1	767-	5.1	3.5-6.7	3.2	1.8-4.4

Total^b^		34.2		31.5			34.2		31.5	

^a^ Minimum values of RFPC and IEN per quintile. The maximum value per quintile corresponds to the minimum value of the next quintile.

^b^ Refers to the total number of beneficiaries of the general sample under study.

The evaluation of BFP coverage and focus was performed in 2004, when both the cohort and the program started, and in 2010, when the children were six years old. The results are presented in Table 3, using two eligibility criteria. The first was based on *per capita* income (≤ R$100 in 2004 and ≤ R$140 in 2010, excluding the value of the benefit). The second was based on being in the first quintile of the IEN (belonging to the 20% poorest). According to *per capita* income, there were 29% eligible families in 2004 and 16% in 2010. Using the IEN, the proportion is fixed at 20% by definition.

**Table 3:** Coverage and focalization of the *Bolsa Família* Program in the 2004 Birth Cohort of Pelotas during 2004 and 2010, using two eligibility criteria.

Year	Indicator	Eligibility criteria			

		*Per capita* household income^a^		National Economic Indicator^b^	
	
		%	95%CI	%	95%CI
2004	Coverage	42.8	40.0-45.6	29.2	26.2-32.3
	Focalization	77.9	74.8-81.0	36.8	33.1-40.4
2010	Coverage	70.9	67.1-74.6	63.0	59.5-66.5
	Focalization	32.4	29.8-35.0	36.9	34.2-39.6

^a^ *Per capita* household income (excluding the benefit received) less than or equal to R$100.00 for 2004 and less than or equal to R$140.00 for 2010.

^b^ Families belonging to the first quintile (20.0% poorest) of the National Economic Indicator.

The results of both criteria are less consistent than in the evaluation of population coverage. According to the *per capita*, 42.8% of eligible people were covered by the program in 2004, and 70.9% in 2010. Using the IEN, the coverage was lower, 29.2% and 63.0%, respectively. Contrary to what happened to coverage, we observed a considerable fall in the focus of the program between 2004 and 2010 using the household income criterion - the percentage of eligible people between beneficiaries fell from 77.9% to 32.4%. According to the IEN, however, we observed no changes in focus, which has always been low, 36.8% in 2004 and 36.9% in 2010.

## DISCUSSION

The 2004 Pelotas Birth Cohort is an ideal study for BFP assessment. This cohort monitors children born in 2004, coinciding with the launch of BFP, and has detailed information from families and children, including socioeconomic, demographic, and health-related data. The municipality of Pelotas is the target of a large number of population studies due to its several research groups, being also quite similar to other medium-sized municipalities in the country regarding health indicators, despite being situated at the southernmost part of Brazil^15^.

We found over 32% of the cohort children families to be BFP beneficiaries, equivalent to 2.5 times the total proportion of beneficiaries in the municipality, which is 13% (about 15 thousand families benefiting in a universe of 114 thousand families). This disproportion is easily explained because all the families of the 2004 cohort necessarily include school-age or preschool children.

The increase in the proportion of families benefiting from BFP is well known, but it is interesting to study how this increase occurs in a closed group of families such as the 2004 cohort, from the beginning of the program. We observed a linear increase until 2006, a stabilization period until 2008, and a new increase in 2009. At this point the coverage reached more than 30% of families. More interesting is the assessment of the proportion of beneficiaries by IEN quintiles (or, in a quite similar way, by *per capita* household income quintiles). Instead of an abrupt fall in the percentage of beneficiaries with increased wealth, standard expected in the case of a focalized program that uses income as an eligibility criterion, we verified a smooth and linear reduction, both in 2004 and 2010 (Figure 2). The expansion of the program, with significant increase in the proportion of beneficiaries that occurred between 2004 and 2010, when we observed an increase from 11% to 34%, should have occurred primarily among the poorest. Our result suggests, however, a proportional increase in all groups of wealth. Therefore, in 2010, 33% of the IEN intermediate quintile, or 28% of *per capita* income intermediate quintile, were receiving assistance from the program.

With this type of distribution, the program was not expected to present high focus. We used two different criteria, due to difficulty to define exactly who is eligible or not for the program. The *per capita* household income criterion is closer to that used in practice, although subject to more information error. The household income reported in the 2004 cohort suffers from usual problems of recording income (recall bias, misinformation on the part of the respondent, or even intentional underreporting), all well documented in the literature^3^ ^,^ ^10^. In the case of BFP, considering that income is a criterion, the problem is more critical. Although we do not believe that there is a relevant intentional income underreporting in the 2004 cohort records, it is important to keep in mind that the beneficiaries may be afraid to report their true income, considering the possibility that this information can be passed on to program managers (although the study makes an explicit promise of confidentiality). On the other hand, the IEN, economic indicator based on household assets and on the education of the head of the family^1^, does not rely on reporting income or expenditure, and has been shown to be a good economic classifier in health equity studies^7^. Less subject to information bias, the IEN does not offer an absolute measure of wealth and can only be used to order the families. It is not a measure of current income either, being seen more as a measure of permanent income and, obviously, also subject to misclassification. Its advantage in this analysis is exactly being independent of reported income.

Given the discussion above, estimation of coverage and focus based on both criteria (income and IEN) allows for a more comprehensive assessment of the program performance. From the point of view of coverage, both criteria show a significant increase in the proportion of eligible people supported by the program – from 43% to 71% using *per capita* household income and from 29% to 63% using the IEN. The results differ in the focus assessment. Using household income, 29% and 16% of families were eligible in 2004 and 2010, respectively, and program focus fell from 78% to 32% (percentage of eligible people among the beneficiaries). This reduction in focus suggests that the expansion of the program resulted in the leakage of the benefit to ineligible families. Using the IEN, we have a different picture, in which program focus was already low and remained low, 37%, from 2004 to 2010. This can be partly explained by the number of people considered eligible by the IEN, a smaller group (20%) compared to the 29% using the income criterion. Also, note that there is not an exact correlation between IEN and income, since the each method measures wealth in a different way.

Other studies using data from the PNAD and the Census estimated BFP focus and coverage that suggest, despite variation in point estimates, that both focus (ranging between 51% and 62%) and coverage (ranging between 14% and 78%) are low at national level^22^ ^,^ ^h^. Focalization errors are attributed mainly to the underreporting of income, filling errors, and volatility of household income. When BFP is compared with other programs, such as *Chile Solidario* (Chile) or *Oportunidades* (Mexico), the focus is almost the same^14^, which suggests that it is difficult to avoid leakage of the benefit to ineligible families in any scenario.

Considering the results based on the IEN, which indicates that in 2004 the program included ineligible families, but that were eligible in terms of income, and considering that we obtained the same focalization in both periods, this study corroborates the literature when showing that, using different approaches (with economic classifications not based on income), focus is low since the beginning of the program^18^ ^,^ ^h^. The low focus of BFP, found in this and other studies, could explain, at least in part, the lack of consistency in the results of the impact studies, and the lack of effect of the program in many of these studies. The program impact can be diluted if many beneficiary families do not actually need it.

The participation of the municipality is important in this process because the supervision of conditionalities and the update of program register (CadÚnico) is performed at this level. The municipality degree of organization makes application to the program easier or more difficult, and better update and verification of information can result in better applicant data quality. However, the decision on the eligibility of the beneficiaries lies in the federal sphere (MDS/SENARC/Caixa Econômica Federal).

Because of the considerable value that has been invested annually in the BFP (more than R$20 billion in 2014, according to MDS^17^), and the relatively low monthly average value provided for families (an average of R$107.00 per family in our study), it seems essential to improve the focalization of the program. This would open the possibility of increasing the value of the benefit to those families who are actually in poverty and extreme poverty, without increasing the program cost. It is important to emphasize that these comments are directly applicable to the municipality of Pelotas and, specifically, to the population of the 2004 cohort, and the results can be influenced by the municipal health management as well as by the organization of complementary services to families such as the periodic monitoring of the families of the cohorts.

## Footnotes

a Presidência da República. Objetivos de desenvolvimento do milênio: 4 º Relatório Nacional de Acompanhamento. Brasília (DF): IPEA; 2010. Available from: www.abc.gov.br/api/publicacaoarquivo/555

b Soares S, Osório RG, Soares FV, Medeiros M, Zepeda E. Programas de transferência condicionada de renda no Brasil, Chile e México: impactos sobre a desigualdade. Brasília (DF): IPEA; 2007 [cited 2015 Oct 14]. (Texto para Discussão, n.1293). Available from: http://www.ipea.gov.br/agencia/images/stories/PDFs/TDs/td_1293.pdf

c Soares S, Ribas RP, Soares FV. Focalização e cobertura do Programa Bolsa-Família: qual é o significado dos 11 milhões de famílias? Brasília (DF): IPEA; 2009 [cited 2015 Oct 14]. (Texto para Discussão n.1396). Available from: http://www.ipea.gov.br/portal/images/stories/PDFs/TDs/td_1396.pdf

d The World Bank. Brasil: aspectos gerais. Washington (DC); [cited 2015 Oct 14]. Available from: http://www.worldbank.org/pt/country/brazil/overview

e Santana JA. A evolução dos programas de transferência de renda e o Programa Bolsa Família. Trabalho apresentado no Seminário População, Pobreza e Desigualdade; 5-7 nov 2007; Belo Horizonte, Brasil. Available from: http://www.abep.nepo.unicamp.br/SeminarioPopulacaoPobreza Desigualdade2007/docs/ SemPopPob07_1019.pdf

f Sousa MF, Ximenes DA, Jaime PC, organizadores. Resultados, avanços e desafios das condicionalidades de saúde do Bolsa Família. Brasília (DF): Ministério do Desenvolvimento Social e Combate à Fome, Secretaria de Avaliação e Gestão da Informação; 2014. (Cadernos de Estudos Desenvolvimento Social em Debate, n.17).

g Souza AP, Duarte J, Neves JAS, Oliveira PP, Gadelha SRB. Uma investigação sobre a focalização do Programa Bolsa Família e seus determinantes imediatos. São Paulo: Fundação Getúlio Vargas; 2013. Available from: http://cmicro.fgv.br/sites/cmicro.fgv.br/files/arquivos/WP_13_2013.pdf

h Melo SRS, Sampaio YS. Focalização do Programa Bolsa Família: uma análise de erros de cobertura e vazamentos [palestra]. Vitória/ES, 2012 [cited 2015 Oct 13]. Available from: http://www.sober.org.br/palestra/15/1264.pdf

i Governo Federal. Portal da Transparência. Available from: www.portaldatransparencia.gov.br

j Osório RG, Souza PHGF, Soares SSD, Oliveira LFB. Perfil da pobreza no Brasil e sua evolução no período 2004-2009. Brasília (DF): IPEA; 2011. I(Texto para Discussão, n.1647). Available from: http://www.ipea.gov.br/portal/images/stories/PDFs/TDs/td_1647.pdf
